# Imaging-guided lumbar facet injections: is there a difference in outcomes between low back pain patients who remember to return a postal questionnaire and those who do not?

**DOI:** 10.1007/s13244-012-0178-8

**Published:** 2012-06-07

**Authors:** Stefanie Kremer, Christian W. A Pfirrmann, Juerg Hodler, Cynthia K. Peterson

**Affiliations:** 1 Department of Radiology, Orthopaedic University Hospital of Balgrist, Forchstrasse 340, 8008 Zürich, Switzerland; 2 Department of Radiology, University Hospital, Rämistrasse 100, 8091 Zürich, Switzerland

**Keywords:** Low back pain, Facet joint pain, Lumbar facet joint, Lumbar intra-articular steroid injection, Outcomes

## Abstract

**Objectives:**

To determine whether data obtained from patients returning postal questionnaires accurately reflect how patients receiving imaging-guided lumbar facet injections respond.

**Methods:**

Seventy-eight patients receiving lumbar facet joint injections who returned an outcomes questionnaire (responders) were age and gender matched with 78 patients who did not return the postal questionnaire (non-responders) after facet joint injections. Baseline numerical rating scale (NRS) pain data were collected. NRS and Patients’ Global Impression of Change (PGIC) data were collected 1 month after injection by postal questionnaire or telephone interview.

Differences in NRS scores were calculated using the unpaired t-test. One level injection patients were compared to patients having ≥2 levels injected using the paired and unpaired t-test. The proportion of patients reporting significant improvement in each group was calculated.

**Results:**

NRS scores were significantly improved compared to baseline (p = 0.0001). Thirty-eight percent of responders were significantly improved compared to 50 % of non-responders. Patients having ≥2 levels injected reported significantly higher baseline NRS scores, but by 1 month there was no difference in NRS scores between groups.

**Conclusions:**

Patients returning postal questionnaires report a less favourable outcome. Telephone interview patients having injections at more than one level have better outcomes.

***Main messages*:**

• *Patients returning postal questionnaires report worse outcomes after facet injection.*

• *Method of data collection should be considered when reporting treatment outcomes.*

• *Patients receiving facet injections at more than one level report greater levels of pain reduction.*

## Introduction

Lumbar facet joints are considered to be a common source of chronic low back pain [1–12]. Golthwait is credited as the first to describe pain originating from the facet joints in 1911 as cited by van Kleef et al. [1], while Ghormley first coined the term “facet syndrome” in 1933 (as cited by van Kleef et al.) [1]. Manchikanti et al. [2] demonstrated that the facet joint is a source of pain in a significant number of patients suffering from chronic low back pain, with further research support for the existence of facet joint pain coming from Berven et al. [3]. Although the presence of a “facet syndrome” has long been questioned, it is now generally accepted as a clinical entity [1]. In accordance with criteria established by the International Association for the Study of Pain, the facet joints have been shown to be the source of chronic low back pain in 15 % to 45 % of patients [4].

Unfortunately, it is not always possible to pinpoint the exact structure or pathology responsible for LBP, because no physical examination findings are pathognomonic for the diagnosis of low back pain of facet origin. Controlled, comparative anesthetic blocks of the lumbar medial branches have been stated to be the most reliable method of diagnosing facet-mediated pain [5]. However, Van Kleef et al. [1] found that both medial branch and intra-articular blocks can be used equally for diagnosis and mentioned that there is no gold standard for diagnosing low back complaints originating from the facet joints.

The treatment of facet pain is also the subject of great controversy. Lumbar facet joint interventions, such as image-guided intraarticular injections, may be used to manage chronic facet-mediated low back pain [6–9]. For carefully selected cases, lumbar facet block is a relatively simple, safe and minimally invasive procedure that can be a valuable adjunct in the treatment [6]. Boswell et al. [7] conclude in their review article that the evidence for lumbar intraarticular facet joint injections for short- and long-term pain relief is moderate for lumbar pain. A more recent systemic review in 2010 [9] also stated that there is moderate to strong evidence supporting the use of injections into the facet joints. Some controversy has been reported for the value of these intraarticular injections in the facet joints in other studies, however. Datta et al. [10] concluded that although there is strong evidence for the diagnostic accuracy of facet joint blocks in evaluating spinal pain, the evidence for therapeutic lumbar intraarticular injections is level III (limited). Furthermore, an earlier study [11] found that intra-articular facet joint injections containing corticosteroids seemed to have no additional therapeutic effect on lower back pain compared to injections of anesthetic alone. It has even been suggested that intra-articular facet joint injections may be no better than placebo for chronic lumbar spine pain [12]. In spite of this conflicting evidence, these injections are commonly used to either diagnose or treat patients with chronic low back pain.

The quality of clinical research depends to a large degree on the validity of data obtained directly from patients. Edwards et al. [13] stated in their systematic review that the response rate of collecting valuable data can be increased by using a shorter questionnaire. Non-response to questionnaires reduces the effective sample size and can introduce bias. Since only half or less than half of patients remember to return questionnaires, it is plausible to ask whether those returned questionnaires truly represent the outcomes from a given procedure. The outcomes comparing questionnaire non-responders to questionnaire responders may give a better idea as to the overall effectiveness of an intervention or procedure. Johansen and Wedderkopp [14] reported that retrospective data can safely be collected for up to 1 month. Beyond that time span, recall becomes imprecise. Another study from Bolton and Humphreys [15] also demonstrated that rating of pain by patients with the Numerical Rating Pain Scale (NRS) provides accurate and reliable information for clinical evaluation and that patients are able to accurately recall their pain retrospectively.

Hundreds of image-guided lumbar facet joint injections are performed every year at our orthopaedic university hospital in Zürich, Switzerland. As part of the quality control evaluation, all patients are handed a short outcomes-based questionnaire collecting pain and quality-of-life data to be returned after 1 month. However, because less than 50 % of patients return these questionnaires and because the outcomes from the patients who do return their questionnaires appeared to be worse than the figures reported in the literature, it was desired to do a comparison of the outcomes from patients who return these questionnaires with those who do not. This is needed to determine if the data from returned questionnaires are an accurate reflection of the true outcomes of patients receiving these lumbar facet joint injections. The purpose of this study was to address this issue.

## Materials and methods

Consecutive patients referred for imaging-guided lumbar facet injections between 5 September 2010 and 22 January 2011 were included in the study. Hospital and regional ethics approval was obtained for all imaging-guided injections and follow-up questionnaire data collection. All patients signed informed consent. A total of 210 patients received these infiltrations, and all were given a postal questionnaire along with a stamped and addressed envelope with instructions to return this completed questionnaire after 1 month. Baseline pain data using the numerical rating scale (NRS, where 0 is no pain and 10 is the worst pain imaginable) were collected on all patients prior to injection as well as at 20–30 min after injection before they left the radiology department. Because only approximately 50 % of the patients returned these 1-month questionnaires, their outcomes were compared to the outcomes of patients who did not return their questionnaires (non-responders) as collected by telephone interview.

### Facet injection procedure

**Fig. 1:**
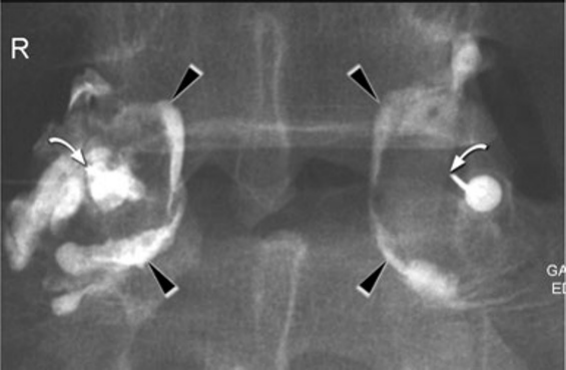
L4-5 bilateral facet injections. Arrowheads show the contrast within the joint capsules. Curved arrow shows the tip of the needle

Under sterile conditions (3× disinfection, sterile gloves, mask, sterile covering), fluoroscopy-guided puncture of the relevant facet joint/s was performed. Documentation of the needle position was done with contrast medium and fluoroscopy. Injection of 40 mg Kenacort (triamcinolone acetonide; Dermapharm AG, Huenenberg AG, Switzerland) and 1 ml ropivacaine (Naropin; Astra-Zeneca, Södertälje, Sweden) was then performed (Fig. 1).

### Outcomes

Baseline and 1-month data were collected for both postal responders and non-responders. For the patients who returned their questionnaire, this data consisted of the patient’s global impression of change (PGIC) assessed at 1 day, 1 week and 1 month after injection of anesthetic plus corticosteroid. It also evaluated the pain response (NRS) 1 day, 1 week and 1 month after injection. The PGIC consists of a 7-point scale anchored to statements about change in the condition from 1 (much better) to 7 (much worse). Only numbers 1 and 2 (much better or better) counted as improvement. ‘Slightly better’ did not count as improvement. A study from Newell et al. [16] showed that change scores in outcome measures correlated with the patient's perception of global improvement (PGIC). They found that the PGIC can be used as a gold standard in categorising patients as improved (or not).

For those patients who did not return their questionnaire (non-responders), only the 1-month PGIC and NRS data were collected by telephone interview no longer than 1 week after the 1-month date for returning the postal questionnaires. The first author checked the imaging database regularly to monitor which patients returned questionnaires and which did not in order to time the telephone calls to the non-responders as close to 1 month after injection as possible. Thus the maximum time that the non-responders had to retrospectively recall their 1-month pain and functional status was 1 week. Therefore, this is a prospective outcomes study on the cohort of patients who returned their questionnaire but a retrospective study using recall of the 1-month data on the same outcomes for the cohort who did not return their postal questionnaire.

Patients who did not return their questionnaires (non-responders) were then age and gender matched (±2 years) to patients who did return their questionnaires (responders). This resulted in 78 patients in each group. Age and gender matching was done blinded to any clinical outcomes.

### Statistical analysis

Data from responders and non-responders were entered into SPSS version 17.0 for analysis. Data were analysed before and after age and gender matching. Age and gender matching was performed because a higher proportion of female patients remembered to return their postal questionnaires. Descriptive statistics were calculated for patient ages, gender distribution, proportion of patients returning their questionnaires and the proportion of patients having only one spinal level injected compared to more than one spinal level injected. The decision to compare patients with one level injected to those with more than one level injected was made based on the literature stating the difficulties in isolating specific structures as the source of low back pain [5, 12] as well as the fact that this was not done in previous studies. Comparisons of the 20–30-min and 1-month NRS scores to the baseline NRS scores were done for both the responders and non-responders combined using the paired Student’s t-test. Gender differences were evaluated for age, the baseline, 20–30-min and 1-month NRS scores using the unpaired t-test.

Differences in NRS scores between the responders and non-responders before and after age and gender matching were calculated using the unpaired t-test. Comparisons of the 20–30-min and 1-month NRS scores to the baseline NRS score were done for each group separately using the paired t-test. Additionally, the proportion of patients responding favorably or unfavorably (PGIC scores) was calculated for each group. Outcomes from patients having only one level injected were compared to those of patients having two or more levels injected using both the paired and unpaired Student’s t-test to evaluate differences within and between groups. Significance was set at p < 0.05.

## Results

Between the dates of 5 September 2010 and 22 January 2011, 210 patients received imaging-guided lumbar facet joint infiltrations. Of these, 45 % of the patients were male and 55 % were female. Only 107 patients returned their postal outcomes questionnaire (responders) (51 %). From the subgroup of non-responders (103 patients), 82.5 % (85 patients) could be reached by telephone, and their 1-month outcome data were collected. Age (±2 years) and gender matching was accomplished for 156 patients (78 responders and 78 non-responders).

Forty-seven percent of all combined (postal responder and non-responder) patients had one level injected compared to 53 % who had two or more levels injected. All patients were treated with therapeutic injections (anesthetic plus corticosteroid). Only one patient called by telephone mentioned a mild adverse reaction (headache).

The mean age of all patients (postal responders and non-responders) was 63.3 years (SD 12.6), with the youngest patient being 23 years old and the oldest patient being 90 years old. There was a significant age difference (p = 0.04) between males and females in the subgroup of the patients who did return their questionnaires. The mean age of the women was 60.8 (SD 13.2) and of the men 64.3 (SD 12.0). In the subgroup of the non-responders there was no significant age difference between females and males.

The baseline NRS pain scores, the 20-30-min NRS scores and the 1-month NRS scores showed no significant gender differences between the responder and the non-responder-groups. The mean NRS baseline pain score of all patients was 7.0 (SD 2.2), the mean NRS score after 20-30 min was 4.0 (SD 2.6) and the NRS score at 1 month after treatment was 4.7 (SD 2.7). The 20-30-min and 1-month NRS scores were statistically significantly improved compared to the baseline score (p = 0.0001) (Table 1).

**Table 1:** NRS results for all patients up to 1 month after injection

Baseline data and outcomes	Mean + (SD)
Pre NRS (baseline)	7.0 (2.2)
20-30-min NRS	4.0 (2.6)*
1-month NRS	4.7 (2.7)*

*p = 0.0001 compared to the baseline score

The results from the Patient’s Global Impression of Change (PGIC) scores prior to age and gender matching indicate that patients who did not return their questionnaires seem to have better outcomes after facet joint injections compared to patients who did return their questionnaires. Of the patient group of responders (questionnaire returned), 33 % were clinically significantly improved at 1 month compared to 49 % of the patients who did not return their postal questionnaire (telephone interviews). Furthermore, 15 % of the patients who did return their postal questionnaire stated that they were worse at 1 month (8 % slightly worse, 5 % worse and 2 % much worse). Only 4 % of the telephone interview patients reported that they were slightly worse, with none of the patients stating that they were worse or much worse (Table 2). This was significantly different at p = 0.02. The remainder of patients reported being unchanged.

**Table 2:** PGIC results for postal patients (responders) and telephone patients (non-responders)

Baseline data and outcomes	Responders (postal)	Non-responders (telephone)
PGIC improved (1 and 2)	33 %	49 %
PGIC worse (5, 6 and 7)	15 %	4 % p = 0.02
Slightly worse (5)	8 %	4 %
Worse (6)	5 %	0 %
Much worse (7)	2 %	0 %

Comparing patients with one level injected to those having more than one spinal level injected when both responders and non-responders were analysed together showed that patients having more than one level injected had higher baseline NRS scores. However, when analysing the two groups separately, this was only statistically significantly different in the telephone interview patients (p = 0.012) (Table 3), whereas it did not reach statistical significance in the responders (postal questionnaire) group (Table 4). The telephone interview patients (non-responders) who had more than one level injected also had significantly higher NRS scores at 20-30 min compared to those patients having only 1 level injected (p = 0.04), but by 1 month there was no significant difference between the two groups. Therefore, particularly non-responder patients having more than one level injected tended to present with higher pain levels, but by 1 month there was no difference compared to patients having only one level injected.

**Table 3:** NRS score results of patients with one level injected compared to those having two or more levels injected within the group of non-responders (telephone patients)

Baseline data and outcomes	1 level injected, mean + (SD)	2 or more levels injected, mean + (SD)	1 vs. 2 level statistical significance (p)
Pre NRS (baseline)	6.5 + (2.4)	7.9 + (2.2)	0.012 **
20-30-min NRS	3.5 + (2.7)*	4.8 + (2.7)*	0.04**
1-month NRS	5.3 + (3.0)*	5.3 + (2.5)*	0.924

*p < 0.05 compared to baseline scores, **p < 0.05 compared to each other

**Table 4:** NRS score results of patients with one level injected compared to those having two or more spinal levels injected within the group of responders (postal questionnaire)

Baseline data and outcomes	1 level injected, mean + (SD)	2 or more levels injected, mean + (SD)	1 vs. 2 level statistical significance (p)
Pre NRS (baseline)	6.8 + (2.1)	7.0 + (2.0)	0.666
20-30-min NRS	3.8 + (2.5)*	4.0 + (2.7)*	0.524
1-month NRS	4.6 + (2.7)*	4.4 + (2.6)*	0.682

*p < 0.05 compared to baseline score

### Age- and gender-matched comparisons

In both patient groups (postal responders and non-responders) the 20-30-min NRS scores and the 1-month NRS scores were statistically significantly improved compared to the baseline score (p = 0.0001). Comparing the baseline NRS scores, the 20-30-min NRS scores and the 1-month NRS scores between the postal patients and the telephone patients showed that the telephone patients had a significantly higher mean 1-month NRS score (p = 0.008) (Table 5)

**Table 5:** NRS results for the age- and gender-matched postal patients (responders) and the telephone patients (non-responders)

Baseline data and outcomes	Responders (postal), mean + (SD)	Non-responders (telephone), mean + (SD)	Responders vs. non-responders, statistical significance (p)
Pre NRS (baseline)	6.8 (2.1)	7.2 (2.4)	0.223
20-30-min NRS	3.7 (2.6)*	4.3 (2.8)*	0.195
1-month NRS	4.2 (2.6)*	5.3 (2.7)*	0.008**

*p = 0.0001 compared to baseline score

**p = 0.008 compared to each other

The PGIC results obtained by age and gender matching of the patients reflect the outcomes calculated from the population that was not age and gender matched. Patients who did not return their questionnaires also reported better outcomes after facet joint injections than patients who did return their questionnaires. Of the patient group of responders (questionnaire returned), 38 % were clinically significantly improved at 1 month compared to 50 % of the patients who did not return their postal questionnaire (telephone interviews). Furthermore, 14 % of the patients who did return their postal questionnaire stated that they were worse at 1 month (6 % slightly worse, 5 % worse and 3 % much worse). Only 4 % of the telephone interview patients reported that they were slightly worse, with none of the patients stating that they were worse or much worse (Table 6). This was statistically significant at p = 0.02.

**Table 6:** PGIC results for age- and gender-matched postal patients (responders) and telephone patients (non-responders)

Baseline data and outcomes	Responders (postal)	Non-responders (telephone)
PGIC improved (1 and 2)	38 %	50 %
PGIC worse (5, 6 and 7)	14 %	4 % P = 0.02
Slightly worse (5)	6 %	4 %
Worse (6)	5 %	0 %
Much worse (7)	3 %	0 %

Comparing the age- and gender-matched patients who had only one level injected to those having more than one spinal level injected for the responders and non-responders together showed that patients having two or more spinal levels injected reported significantly higher mean baseline NRS scores (7.6, SD = 2.2) compared to those having only one level injected (6.5, SD = 2.3, p = 0.005). This difference remained statistically significantly higher in the multiple injection level group when looking at the telephone interview patients alone (p = 0.03) (Table 7). However, it did not reach statistical significance in the responder (postal questionnaire) group (Table 8). The telephone interview patients (non-responders) who had more than one level injected still had higher NRS scores at 20-30 min compared to those patients having only one level injected (p = 0.053), but this did not quite reach statistical significance. At 1 month there was no significant difference between the two groups. Therefore the trend is for patients having two or more levels injected to present with more pain, but by 1 month post injection to have improved such that there is no difference in pain level compared to patients having only one level injected.

**Table 7:** NRS score results of age- and gender-matched patients with one level injected compared to those having more than one spinal level injected within the group of non-responders (telephone patients)

Baseline data and outcomes	1 level injected, mean + (SD)	2 or more levels injected, mean + (SD)	1 vs. > 2 levels (p)
Pre NRS (baseline)	6.5 + (2.5)	7.8 + (2.3)	0.03
20-30-min NRS	3.5 + (2.9)*	4.8 + (2.7)*	0.053
1-month NRS	5.4 + (3.1)*	5.2 + (2.5)*	0.765

*p < 0.05 compared to the baseline score

**Table 8:** NRS score results of age- and gender-matched patients with one level injected compared to those having more than one spinal level injected within the group of responders (postal questionnaire)

Baseline data and outcomes	1 level injected, mean + (SD)	2 or more levels injected, mean + (SD)	1 vs. > 2 levels (p)
Pre NRS (baseline)	6.5 (2.1)	7.2 (2.1)	0.144
20-30-min NRS	3.7 (2.6)*	3.8 (2.6)*	0.991
1-month NRS	4.4 (2.5)*	3.9 (2.7)*	0.371

*p < 0.05 compared to baseline scores

## Discussion

The purpose of this study was to determine whether or not the outcome data obtained from patients returning postal questionnaires are an accurate reflection of how patients receiving image-guided lumbar facet injections respond. At least at this orthopaedic university hospital patients who return their outcomes postal questionnaires report that they are less likely to experience clinically relevant improvement and significantly more likely to be worse 1 month after lumbar facet injections compared to patients who forgot to return their postal questionnaires but were interviewed via telephone. Therefore, relying only on the data obtained from the postal questionnaires gives a less favourable impression of the outcomes from this intervention. This is not too surprising as it is logical that patients who are still suffering or particularly those who are worse after injection desire to provide feedback. Indeed, several of the envelopes containing the returned postal questionnaires also included hand-written letters with specific details about the patient’s pain and suffering.

This current study supports the findings from previous research in that patients receiving lumbar intra-articular facet joint injections reported significantly reduced mean low back pain NRS scores [6–9] and many patients also reported clinically significant overall improvement (PGIC). The mean NRS scores of both postal responder and non-responder patients at 20-30 min and 1 month after injection were statistically significantly improved compared to their mean baseline scores.

Interestingly, in the age- and gender-matched population, those patients who received the telephone interview (i.e. did not return their questionnaire) had a significantly higher mean 1-month NRS score compared to the patients returning their postal questionnaire, yet 50 % of the telephone interview patients reported according to their PGIC score that they were clinically significantly improved compared to only 38 % of the postal (responders) patients. The NRS score only covers the pain intensity of low back pain, whereas the PGIC also contains psychological domains that may be influential, especially in chronic conditions. Newell and Bolton [16] showed that even at 4 weeks psychosocial components are already exerting significant effects on the success of treatment. Thus, the PGIC is considered more of a gold standard in categorising patients as improved or not.

Patients having more than one lumbar facet level injected started with significantly higher mean pain scores compared to patients having only one level injected. However, at 1 month there was no significant difference between the two groups in their mean NRS scores. This was particularly true for the non-responder group. Thus patients who had injections at more than one spinal level seemed to obtain a greater magnitude of pain reduction. A potential reason for this is the difficulty in identifying one specific generator of low back pain, particularly for diagnosing the facet joints as a source of low back pain. There is no radiographic finding, characteristic physical examination or specific clinical symptom that proves lumbar facet-mediated pain with certainty [1, 3]. The radiographic severity of facet disease is also not associated with pain severity among those with chronic low back pain [17]. Even magnetic resonance imaging scans of the lumbar spine are unable to predict the development of low-back pain [18]. Therefore, injecting at more levels provides a higher chance of targeting the suspected painful facet joints, particularly in those patients whose pain arises from more than one facet articulation. Although both groups were age and gender matched, this difference in the mean baseline NRS scores between the two groups clearly shows that they were not matched for all factors.

The validity of any outcome data however depends to a large degree on the ability of patients to accurately recall their pain levels, disability and other quality-of-life measures. This is especially important when the recall of pain intensity is done retrospectively. It has been shown from different studies that patients are able to fairly accurately recall and rate an average of their pain intensity across a number of lengths of time, from 24 h [19] to 1 week [15] even up to 1 month [14]. Thus, it is likely that the outcome data from the patients receiving telephone calls in this study accurately reflect the pain severity and overall feeling experienced by these patients during the inquiry time period. This is particularly true as the 1-month telephone calls, although retrospective data collection, occurred no longer than 1 week after the 1-month questionnaire was due. This is well within the time period for accurate recall of data [14, 15].

Another important influence on the accuracy/validity of outcome data is the form of data collection used. Several methods exist for collecting data, including hard copy questionnaires, on-line questionnaires (including text messaging), face-to-face interviews and telephone interviews. The findings of different studies imply that a mode-of-administration effect exists [20–22]. The interest and understanding of a live interviewer may encourage more positive responses, while the anonymity provided by mail surveys may lead to more accurate reports [22]. In general, it is advisable to take the mode of data collection into account when selecting values for comparison. Researchers should consider this when comparing results from similar studies that use different methods for collecting the data [20–22]. As noted above, patients who were interviewed by telephone reported better overall 1-month outcomes (PGIC) after facet joint injections compared to patients who did return their questionnaires and were also much less likely to report that they were worse after injection. The effect of the method of administration or type of data collection therefore may have influenced these results (i.e. postal questionnaire vs. telephone interview) [20–22]. These differences between postal patients (responders) and telephone interview patients (non-responders) are consistent with previous research in which telephone administration yielded more positive reports than self-administration [20–22]. However, the fact that the telephone interview patients in this current study had higher 1-month NRS pain scores compared to those who returned their questionnaire, thus reporting higher pain levels, suggests that these telephone data are likely to be reliable and valid. Interview administration (by telephone) also yields higher participation rates (82.5 % could be reached) than administration by questionnaires (only 51 % returned their completed questionnaire). It was particularly noticed during the telephone interview that many of the patients who did not return their questionnaires were often foreign nationals and therefore may not have had sufficient German reading and comprehension skills to complete the questionnaire. This could also be a reason for non-response.

The main limitation of this study is also related to the main purpose of the study, to determine if outcomes from lumbar facet injection depend on the method of data collection. During the telephone interview, the patients might have a tendency to try and please or impress the interviewer, and therefore may have been reluctant to be totally honest. Most of the patients called by telephone were positively surprised at receiving the telephone call from the hospital inquiring about their response to the injections. Therefore, many patients were in a positive mood while they were answering the questions, which could have boosted their answers. However, the fact that the mean NRS pain scale results for the telephone patients demonstrated higher pain levels after 1 month compared to the postal patients suggests that the results obtained in this study, at least for the NRS data, are likely accurate [15].

Another limitation to this study is the relatively short follow-up data collection time period of 1 month. However, it is common for patients at this specialised orthopaedic/rheumatology hospital to present for subsequent injections and it was desired that the patients included in this study had only one injection during the data collection period so that more accurate comparisons between postal questionnaire and telephone interview methods of data collection could occur. Perhaps the true outcomes from lumbar facet injections lie somewhere between the poorer results reported by those returning questionnaires and the better results from patients reporting by telephone.

## Conclusion

In conclusion, the findings of this study showed that patients who did not return their questionnaires (and thus were interviewed by telephone) reported clinically significantly better overall outcomes after lumbar facet joint injections compared to patients who did return their questionnaires. Therefore, relying only on the results of patients returning the postal questionnaire would have given a less favourable impression of the outcomes from these injections. Patients who had injections at more than one spinal level, particularly those who did not remember to return their questionnaire, seemed to obtain greater improvement in pain reduction. These results may be relevant to other research in which outcomes are assessed using questionnaires.
